# Genetic associations with micronutrient levels identified in immune and gastrointestinal networks

**DOI:** 10.1007/s12263-014-0408-4

**Published:** 2014-05-31

**Authors:** Melissa J. Morine, Jacqueline Pontes Monteiro, Carolyn Wise, Candee Teitel, Lisa Pence, Anna Williams, Baitang Ning, Beverly McCabe-Sellers, Catherine Champagne, Jerome Turner, Beatrice Shelby, Margaret Bogle, Richard D. Beger, Corrado Priami, Jim Kaput

**Affiliations:** 1 The Microsoft Research – University of Trento Centre for Computational and Systems Biology (COSBI), Rovereto, Italy; 2 Department of Mathematics, University of Trento, Trento, Italy; 3 Department of Pediatrics, Faculty of Medicine, Nutrition and Metabolism, University of São Paulo, Ribeirão Preto, SP Brazil; 4 Division of Personalized Nutrition and Medicine, National Center for Toxicological Research, Food and Drug Administration (NCTR/FDA), Jefferson, AR USA; 5 Division of Systems Biology, National Center for Toxicological Research, Food and Drug Administration, Jefferson, AR USA; 6 Delta Obesity Prevention Research Unit, United States Department of Agriculture – Agricultural Research Service, Little Rock, AR USA; 7 Dietary Assessment and Nutrition Counseling, Pennington Biomedical Research Center, Baton Rouge, LA USA; 8 Boys, Girls, and Adults Community Development Center & the Phillips County Community Partners, Marvell, AR USA; 9 Systems Nutrition and Health Unit, Nestlé Institute of Health Sciences, Lausanne, Switzerland

**Keywords:** Systems nutrition, Community-based participatory research, Genetic analysis, Network analysis

## Abstract

The discovery of vitamins and clarification of their role in preventing frank essential nutrient deficiencies occurred in the early 1900s. Much vitamin research has understandably focused on public health and the effects of single nutrients to alleviate acute conditions. The physiological processes for maintaining health, however, are complex systems that depend upon interactions between multiple nutrients, environmental factors, and genetic makeup. To analyze the relationship between these factors and nutritional health, data were obtained from an observational, community-based participatory research program of children and teens (age 6–14) enrolled in a summer day camp in the Delta region of Arkansas. Assessments of erythrocyte *S*-adenosylmethionine (SAM) and *S*-adenosylhomocysteine (SAH), plasma homocysteine (Hcy) and 6 organic micronutrients (retinol, 25-hydroxy vitamin D3, pyridoxal, thiamin, riboflavin, and vitamin E), and 1,129 plasma proteins were performed at 3 time points in each of 2 years. Genetic makeup was analyzed with 1 M SNP genotyping arrays, and nutrient status was assessed with 24-h dietary intake questionnaires. A pattern of metabolites (met_PC1) that included the ratio of erythrocyte SAM/SAH, Hcy, and 5 vitamins were identified by principal component analysis. Met_PC1 levels were significantly associated with (1) single-nucleotide polymorphisms, (2) levels of plasma proteins, and (3) multilocus genotypes coding for gastrointestinal and immune functions, as identified in a global network of metabolic/protein–protein interactions. Subsequent mining of data from curated pathway, network, and genome-wide association studies identified genetic and functional relationships that may be explained by gene–nutrient interactions. The systems nutrition strategy described here has thus associated a multivariate metabolite pattern in blood with genes involved in immune and gastrointestinal functions.

**Electronic supplementary material:**

The online version of this article (doi:10.1007/s12263-014-0408-4) contains supplementary material, which is available to authorized users.

## Introduction

Phenotypic differences between individuals result from heterogeneous genetic makeups sharing the same environment and between genetically similar individuals exposed to different environments. For example, the incidences of obesity and related metabolic disorders among ancestral groups sharing the same environment differ (Ramos and Rotimi 2009; Bustamante et al. 2011) while Pima Indians have different incidences of obesity in Mexico versus the United States (Schulz et al. 2006). A large number of studies have identified gene–environment interactions based on single-nucleotide polymorphisms (SNPs) and nutrient intake (Fenech et al. 2011; Lee et al. 2011; Ordovás et al. 2011), and recent genome-wide association studies (GWAS) have identified SNPs associated with dietary preference (Hamza et al. 2011; Tanaka et al. 2013). These gene–nutrient association studies and GWAS identified individual SNPs that explain only a small fraction of the phenotype (i.e., small effect size) (Goldstein 2009). The focus on individual SNPs, copy number variations (CNVs), or other single genomic structural variations (e.g., insertion/deletions or INDELS) is based implicitly on the one gene–one enzyme hypothesis of Beadle and Tatum (Beadle and Tatum 1941). Their experimental paradigm revolutionized biomedical research by demonstrating that a mutation in a single gene could eliminate enzyme activity and produce a change in phenotype. However, they described biological processes more holistically in the introduction of that landmark paper:

“…Since the components of such a [*sic*] system are likely to be interrelated in complex ways, and since the synthesis of the parts of individual genes are presumably dependent on the functioning of other genes, it would appear that there must exist *orders of directness of gene control ranging from simple one*-*to*-*one relations to relations of great complexity”* (Beadle and Tatum 1941). (Emphasis added)

Systems thinking and methodologies hold greater promise in understanding the complex phenotypes of chronic disease or response to nutrients in foods than the focus on individual genetic variants or the identification of independent environmental factors (Patel et al. 2010, 2012a, b) that influence biological processes. An increasing number of reports employ systems designs and analysis of high-dimensional data from studies of obesity, cardiovascular, nutrition, diabetes, drug, toxicology, immunology, gut microbiota, medicine, health care, and health disparities (Slikker et al. 2007; Auffray et al. 2009; Gardy et al. 2009; Kalupahana and Moustaid-moussa 2011; Kleemann et al. 2011; Roux 2011; Karlsson et al. 2011; Afacan et al. 2012; Meng et al. 2013).

With the exception of several publications that include dietary intake variables as a part of omics-based systems (Morine et al. 2010, 2011, 2012) or genomic analysis (Nettleton et al. 2010), many systems studies have implicitly analyzed biological processes as closed systems since environmental variables were not included in the analysis. Biological processes occur in open systems (Von Bertalanffy 1950), and ex vivo factors, which include nutrients and other naturally occurring chemicals in food, can alter biochemical processes and signaling networks occurring within the organism (Kaput and Rodriguez 2004). Excluding external factors that influence internal biological processes generates an incomplete system at best, likely an inaccurate understanding of the interactions between environment and genetic makeup, and from a practical standpoint, misses an opportunity to identify modifiable factors that influence health.

This report details the design and conduct of a discovery-based pilot study that accounts for (1) the known genetic uniqueness of individual humans (Olson 2012), (2) the intra-individual variability in homeostatic measurements (Williams 1956; Illig et al. 2010; Suhre et al. 2011), and (3) the challenge of characterizing complex phenotypes resulting from small contributions of many genetic and environmental factors (Goldstein 2009). The participants in the Delta Vitamin Obesity intervention study were children and teens (age 6–14) enrolled in a summer day camp that was a component of a community-based participatory research (CBPR) program. CBPR is a form of translational research that engages the participant, members of the community, and scientists in research, education, and health-promoting activities for improving personal and public health (McCabe-Sellers et al. 2008). A detailed description of the intervention and results obtained by aggregating data from individuals for population-level analysis such as metabolite and protein variation in relation to BMI, sex, and age has been reported (Monteiro et al. 2014).

In this report, analysis of the data from the Delta Vitamin Obesity (Monteiro et al. 2014) is extended to further characterize metabolite–metabolite interactions with discovery-based methods that identify systems-wide relationships between metabolites, proteins, nutrient intakes, and genetic makeup. Principal component analysis (PCA) was used to analyze plasma homocysteine (Hcy); vitamins A, D, E; riboflavin; thiamine; pyridoxal; and erythrocyte *S*-adenosylmethionine and *S*-adenosylhomocysteine (SAH) metabolites. A quantitative variable (met_PC1) from the PCA was defined and used for discovering metabolite–protein correlations as well as thousands of genotypes associated with met_PC1 values. Two recent studies have used inferences based on heritability and Bayesian approaches to identify thousands of SNPs associated with height and weight (Hemani et al. 2013) and rheumatoid arthritis (Stahl et al. 2012) to demonstrate that complex phenotypes are the result of thousands of SNPs. Subsequent data mining methods associated genes and proteins identified in this report to biological functional classes including predominantly immune and gastrointestinal function. Finally, the challenges of conducting case–control studies in light of genetic and cultural differences within and between populations are discussed.

## Materials and methods

### Participants and CBPR methods

A description of the summer day camp in the Marvell, AR (USA) school district, 24-h dietary intakes, body weight and height, blood sampling and processing, and proteomic and genomic analysis are provided in (Monteiro et al. 2014). In brief, assessments were conducted before the beginning of the camp (baseline), at the end of 5 weeks of the camp (end of camp), and 1 month after camp ended (post-camp). Metabolite and dietary intake data were averaged across the three assessments for the analysis in this study. Thirty-six participants were recruited in year 1, and 19 completed all three assessments. In the second year, 72 participants enrolled and 42 completed three assessments. None of the children or adolescents (age 6–14) was taking prescribed medicines, nor did they have overt malnutrition, active infection, or known genetic disease that could alter metabolism. All participants were healthy African American children and adolescents. Results for the three assessments are reported. The biomedical research protocol was approved by the FDA’s Research Involving Human Subjects Committee (RIHSC) and the University of Arkansas for Medical Sciences (UAMS) Institutional Review Board (IRB).

### HCY

Total Hcy was analyzed in plasma using a Hcy HPLC Kit (ALPCO Immunoassays, Salem, NH) and a UPLC Waters Acquity HSS T3 column (2.1 × 50 mm, 1.8 µm) coupled with an Acquity HSS T3 1.8 µm VanGuard pre-column at 40 °C.

### Lipid-soluble vitamins

Vitamins were determined using LC/MS/MS (NCTR-FDA-USA): 250 µL of plasma, in a 1.5-mL Eppendorf microcentrifuge tube, was spiked with stable isotope-labeled standards and mixed with 740 µL of MeOH. Samples were held at 4 °C for 30 min. About 500 µL of hexane was added, and samples were centrifuged at 13,000×*g* for 12 min (4 °C). The (top) hexane layer was transferred into a total recovery autosampler vial, and the sample was subsequently extracted with two additional 500 µL hexane portions, each time transferring the hexane layer into the autosampler vial. The combined hexane extracts were placed under a stream of nitrogen gas, dried, and reconstituted in 50 µL of 50:50 MeOH/ACN. Ten microlitre sample was injected on an Acquity UPLC equipped with a 2.1 mm × 50 mm (1.7 µm particle) BEH C18 column held at 35 °C. The mobile phase A was 90:10 water/ACN, and the mobile phase B was 50:50 MeOH/can with a flow rate of 0.5 mL/min. Metabolites were analyzed on a Xevo TQ operated in positive APCI ionization mode using the following parameters: source temperature was 145 °C, corona was 15 uA, probe temperature was 575 °C, and desolvation gas flow rate was 600 L/h. Multiple reaction monitoring **(**MRM) was optimized by direct infusion of standards. The transitions monitored for vitamin A were *m*/*z* 269 → 109 (cone *E* = 35 V, collision *E* = 15 V) and *m*/*z* 269 → 93 (cone *E* = 26 V, collision *E* = 14 V). The transition monitored for vitamin E was *m*/*z* 431 → 165 and for (d3) vitamin E was *m*/*z* 434 → 165 (cone *E* = 35 V, collision *E* = 15 V). The transition monitored for 25-hydroxy vitamin D3 was *m*/*z* 401 → 159 and *m*/*z* 407 → 159 for the (d6) 25 hydroxy vitamin D3 with cone and collision energies of 24 and 28 V, respectively.

### Water-soluble vitamins

About 250 µL of plasma was mixed with 1 mL of (4 °C) acetonitrile in a 1.5-mL Eppendorf microcentrifuge tube. The sample was vortexed briefly and then centrifuged at 13,000×*g* for 10 min at 4 °C. The supernatant was transferred to a total recovery autosampler vial, and the solvent was evaporated. Samples were reconstituted in 250 µL of water (Optima grade), and 10 µL of sample was injected onto an Acquity UPLC equipped with an HHS T3 2.1 × 100 mm, (1.8 µm particle) UPLC column. Mass spectrometric detection was performed on a Xevo TQ (Waters) operated in ESI positive mode using the following parameters: source temperature was 150 °C, capillary voltage (kV) was 2.2, desolvation temperature was 400 °C, and desolvation gas flow rate was 800 L/h. MRMs for target analytes were optimized by direct infusion of standards. The transition monitored for pyridoxal was *m*/*z* 168 → 94 (cone *E* = 16 V, collision *E* = 22 V), for pyridoxine *m*/*z* 170 → 134 (cone *E* = 22 V, collision *E* = 22 V), for thiamine *m*/*z* 265 → 122 (cone *E* = 20 V, collision *E* = 12 V), for riboflavin *m*/*z* 377 → 243 (cone *E* = 40 V, collision *E* = 22 V), and for folic acid *m*/*z* 442 → 295 (cone *E* = 22 V, collision *E* = 12 V).

### Red blood cell *S*-adenosyl-l-methionine (SAM) and *S*-adenosyl-l-homocysteine (SAH)

Red blood cell samples stored at −70 °C were randomly assayed in batches of 20. About 600 µL of red blood cells was added to tubes containing 150 µL of ice-cold trichloroacetic acid (40 % w/v), plus 330 µL 0.1 M sodium acetate trihydrate, and then vortexed. Samples were incubated at 4 °C for 30 min, followed by centrifugation at 15,000 rpm for 15 min. About 150 µL of supernatant was filtered using a 0.22-µm filter, spun at 5,000 rpm for 5 min, and transferred to vials for chromatographic analysis of SAM. The remainder of the supernatant was transferred to a clean tube for ether extraction. Samples were extracted twice with 300 µL, and any remaining ether was evaporated under argon before filtration and transferred to UPLC vials for the analysis of SAH. Standards for SAM and SAH were obtained from Sigma (St. Louis, MO). Chromatographic separation was achieved on an Acquity HSS T3 column (2.1 × 50 mm, 1.8 µm) coupled with an Acquity HSS T3 1.8 µm VanGuard pre-column at 40 °C. The peaks were separated isocratically with an elution time of 5.0 min for SAM and 2.0 min for SAH at 97 % A (buffer) and 3 % B (methanol). The buffer composition for SAM was 50 mM potassium phosphate and 10 mM heptane sulfonic salt adjusted to pH 4.38 with phosphoric acid. The composition for the SAH mobile phase was 50 mM potassium phosphate. Column equilibration time required for SAM was 90 min, while equilibration time for SAH was just 30 min at flow rates of 0.575 mL/min. Buffers and solvents are filtered using 0.22-µm filters prior to use. Samples were held at 4 °C for the duration of the analysis. The injection volume for samples and standard was 10 µL. Detection was performed with a photodiode array detector set to monitor wavelengths 210–400. Standard was prepared in a range from 0.78 to 25.00 pmol/µL for SAH and from 0.32 to 10.40 µL for SAM. A standard curve was generated to allow for automated calculation of results using the Waters Empower software.

### Proteomics

The plasma proteome was quantified for 110 samples from 6 different time points (3 in year 1 and 3 in year 2) but data from 61 at time point 1 were used in these analyses due to missing samples. SomaLogic Inc. (Boulder, CO) performed all proteomic assessments and was blinded to the clinical characteristics of participants in this study. Samples were analyzed as previously described (Gold 1995; Brody and Gold 2000; Gold et al. 2010; Ostroff et al. 2010; Brody et al. 2012).

### Genomic analysis

#### DNA preparation

About 1 mL of whole blood sample from each participant was used for DNA extraction. The genomic DNA samples were extracted and purified using the QIAamp DNA Blood Mini Kit (QIAGEN, Valencia, CA), following the protocol provided by the manufacturer. The quality and quantity of each DNA samples were measured using a NanoDrop 8000 (Thermo Scientific, Wilmington, DE). The Infinium Whole Genome Genotyping technology with the HumanOmni1-Quad version 1.0 kits (Illumina, San Diego, CA) was used for genotyping analyses following the manufacturer’s protocol. The arrays were scanned on a high-resolution iScan (Illumina) and processed using the BeadStudio software version 3.1 (Illumina). The overall genotyping call rate on all samples was above 98 %. Data from 45 unique participants (15 participants attended both years) met these criteria.

#### Preprocessing of genotyping data

Raw SNP data were first preprocessed, removing SNPs with a GC score <0.7, and those that were not genotyped in all participants. SNPs with minor allele frequency <0.1 and those significantly diverging from Hardy–Weinberg equilibrium were also removed. The remaining SNPs were filtered to include only those present in the metabolic/protein–protein interaction (PPI) network used in the analysis, resulting in a final dataset of 125,959 SNPs.

#### Network analysis

A metabolic/PPI network was constructed based on the human interaction networks manually curated databases (Ma et al. 2007; Yu et al. 2012). The largest connected component of this network comprised 116,210 interactions between 13,705 genes, containing 125,959 SNPs present on the Illumina 1 M Quad Array. The network was partitioned into topological modules using the spinglass.community function in the igraph library in R (Csardi and Nepusz 2006) resulting in 58 topological modules (mean module size: 236 nodes; SD: 564 nodes).

#### SNP-, gene-, and network-level analyses

Significant correlations between genotype and met_PC1 levels were assessed in each SNP using generalized estimating equations (GEE), as implemented in the geepack library in R (Højsgaard et al. 2006). Met_PC1 was modeled as a function of genotype at each SNP locus, controlling for age, gender, average Healthy Eating Index, and sibling relationships among the participants (the latter being included as an independence correlation structure in the GEE models). Although some participants attended both years of the camp, only one genotype per participant was used in this analysis. Resulting *p* values were corrected for multiple testing using the procedure proposed by Benjamini and Hochberg (1995). Nominal *p* values were used as input for the VEGAS algorithm, which accounts for size, level of polymorphism, and linkage disequilibrium relationships within genes to determine genewise *p* values from SNP-level results (Liu et al. 2010). Genes reaching significance (*q* < 0.1) were used in hypergeometric tests (implemented using the HTSanalyzeR library in R) to determine significant enrichment of each of the 58 modules in the interaction network. Modules with *q* value <0.1 were considered as significantly enriched in genes related to micronutrients. In order to assess the biological processes that may be directly or indirectly implicated by genetic variation in our met_PC1 genes, the functional profile of each significant module was determined using the ClueGO (Bindea et al. 2009) plugin for Cytoscape. ClueGO functional profiles illustrated in Fig. 6 and Supplementary files include KEGG pathways that are significantly overrepresented among module nodes, using hypergeometric tests and correcting *p* values using the Benjamini and Hochberg method (see Bindea et al. 2009) for technical details on the generation of functional profile networks.

Significant genes were also analyzed in the context of the ArrayTrack QTL database (Harris et al. 2009; Xu et al. 2010) to determine significantly overrepresented QTL phenotypes. Gene sets were constructed by combining all genes within 1 Mbp of QTL mapping to each of the 36 phenotypes (containing at least one significant gene from our analysis) in the ArrayTrack database. Hypergeometric tests were then performed to identify which QTL phenotypes were significantly enriched in the significant genes from our analysis.

## Results

### PCA of metabolite levels

**Fig. 1:**
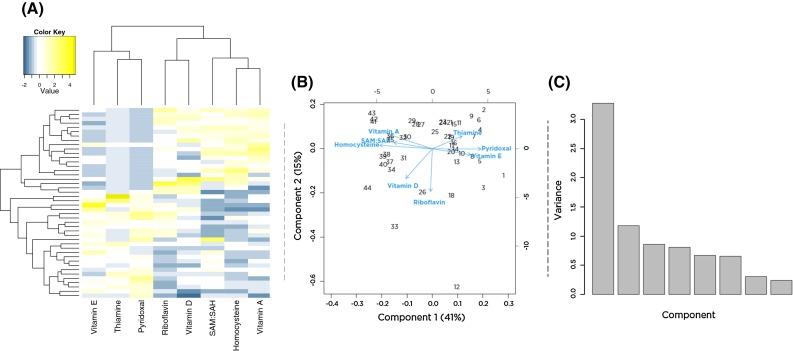
Metabolite-level heat map and principal component analysis of vitamin levels. **a** Metabolite heat map where individuals are represented in the *rows,* and mean value of metabolite levels from three blood samplings is in the *columns*. **b** Principal component analysis of mean values of vitamin or metabolites. *Numbers* indicate values for individuals (**c**). Variances in each principal component (see “Materials and methods” section for details)

Mean plasma levels of metabolites (Hcy; riboflavin; pyridoxal; thiamine; and vitamins A, D, E) and erythrocyte SAM/SAH are illustrated with hierarchical clustering in Fig. 1a. This analysis revealed that individuals (represented in rows) with higher SAM/SAH tended to have higher plasma levels of fat-soluble vitamins A and D and medium or low plasma levels of vitamin E, thiamine, and pyridoxal. Individuals with low SAM/SAH tended to have the opposite patterns of these metabolites.

Given the strong patterns of correlation among the plasma metabolites, PCA was used to identify latent metabolite variables. The first principal component (met_PC1) explained 41 % (Fig. 1b, c) of the variation in metabolite profile and stratified the participants primarily based on their levels of vitamin A, Hcy, SAM/SAH, thiamine, pyridoxal, and vitamin E. Vitamin D and riboflavin contributed to the second principal component and explained 5 % of the variation (Fig. 1b, c) in the dataset. To our knowledge, these nutrient–nutrient associations have not been previously reported and would not have been identified by standard single-variant analysis. Although met_PC1 is a continuous variable, the analysis and heat map indicate metabolic patterns that could be used to group individuals for different nutritional interventions.

### Proteomic associations with metabolite patterns

**Fig. 2:**
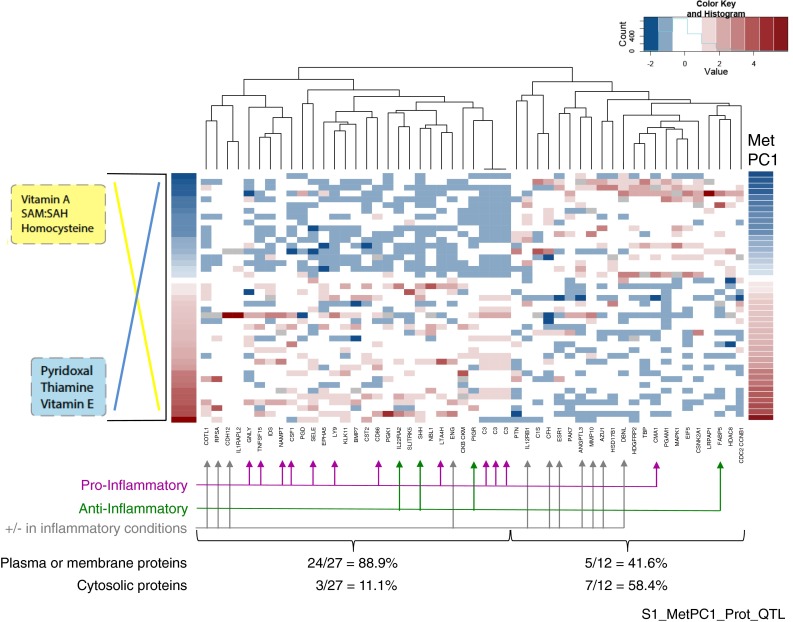
Proteins significantly correlated with met_PC1 (corrected *p* value <0.1). Participants are represented in heat map rows, ordered from low met_PC1 (higher vitamin A, SAM/SAH, homocysteine) to high met_PC1 (higher pyridoxal, thiamine, vitamin E) as shown on the *left* of the figure. Individual proteins were annotated using OMIM or published literature for role in inflammation. Proteins expected to be in the plasma or on membranes exposed to the plasma were identified. *Brackets* at the *bottom* of the figure correspond to the two main branches of the heat map. Plasma and membrane proteins to cluster to the *left* (24/27 = 88.9 %) compared to the *right* branch (5/12 = 41.6 %)

Plasma levels of 1,129 proteins in baseline samples were analyzed using SomaLogic DNA aptamer technology (Kraemer et al. 2011; Gold et al. 2011). Robust linear regression identified 51 proteins significantly associated with the met_PC1 variable at *p* < 0.1 corrected for multiple comparisons (Fig. 2; Supplement 1). Values of met_PC1 corresponding to high vitamin A, Hcy, SAM/SAH were negatively correlated with well-studied proinflammatory proteins, such as CSF1, TNFSF15, C3, SELE, and CD86. These proteins had a weak positive correlation with met_PC1 corresponding to high levels of vitamin E, pyridoxal, and thiamine.

Correlation analysis and hierarchical clustering produced two main branches differing in the percentage of plasma-soluble and membrane proteins versus cytosolic proteins (Fig. 2; see brackets at bottom). We previously observed two clusters of blood versus cytosolic proteins associated with erythrocyte SAM/SAH ratios (Monteiro et al. 2014). The cytosolic proteins in the blood were likely produced by apoptotic processes, although the current data cannot discriminate between normal and pathological cell death.

### Genetic analyses

#### Analysis of genotype–metabolite correlations within a global protein interaction network

**Fig. 3:**
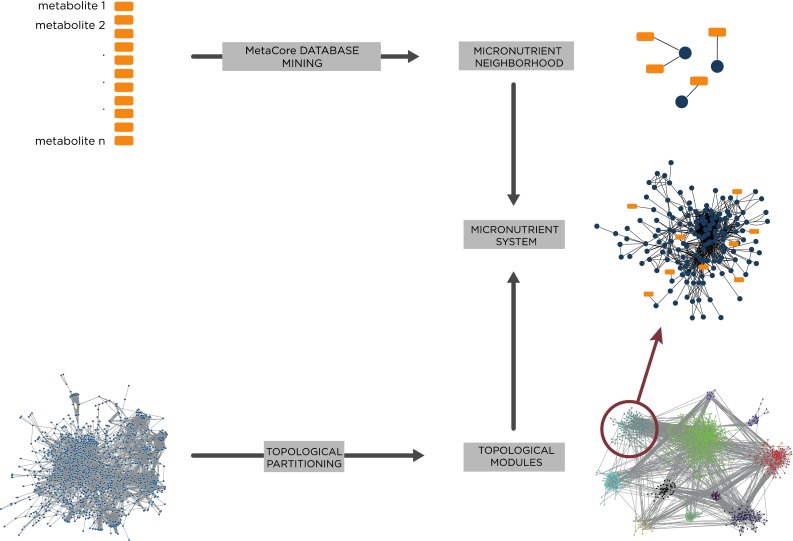
Conceptual approach for gene selection. The analysis focused on SNPs within genes with known functional association with the metabolites (*orange rectangles*) measured in the study. This was accomplished by mining all genes from the MetaCore database with a direct functional connection to any of these metabolites. This resulted in 275 unique genes, designated as micronutrient neighbor genes (*black balls*). In parallel, a global metabolic/PPI interaction network was partitioned into topological modules. Modules that were significantly enriched in micronutrient neighborhood genes were defined as micronutrient systems

Micronutrients and their associated metabolites are involved in a larger network of interactions than can be identified by metabolic pathway tools or pairwise regulatory or protein–protein interactions. The Edinburgh human metabolic network and a second manually curated interaction database (Ma et al. 2007; Yu et al. 2012) were used to construct a global network of metabolic and protein–protein interactions (Fig. 3). The largest connected component of this network comprised 116,210 interactions between 13,705 genes. This global network was subsequently partitioned into topological modules using a heuristic approach to topological partitioning based on simulated annealing (Reichardt and Bornholdt 2006). The resulting global network consisted of 58 topological modules with mean module size of 236 nodes (minimum module size: 2; maximum size: 2351). Topological modules are loosely analogous to biological pathways in that they represent functionally cohesive groups of interacting genes/proteins, and as such they provide a partitioning of the overall network into biological subsystems to be used as a framework for the omics data analysis. The advantage of network modules as an alternative to pathways is that they capture the inherent overlap and intersection between canonical pathway models.

**Fig. 4:**
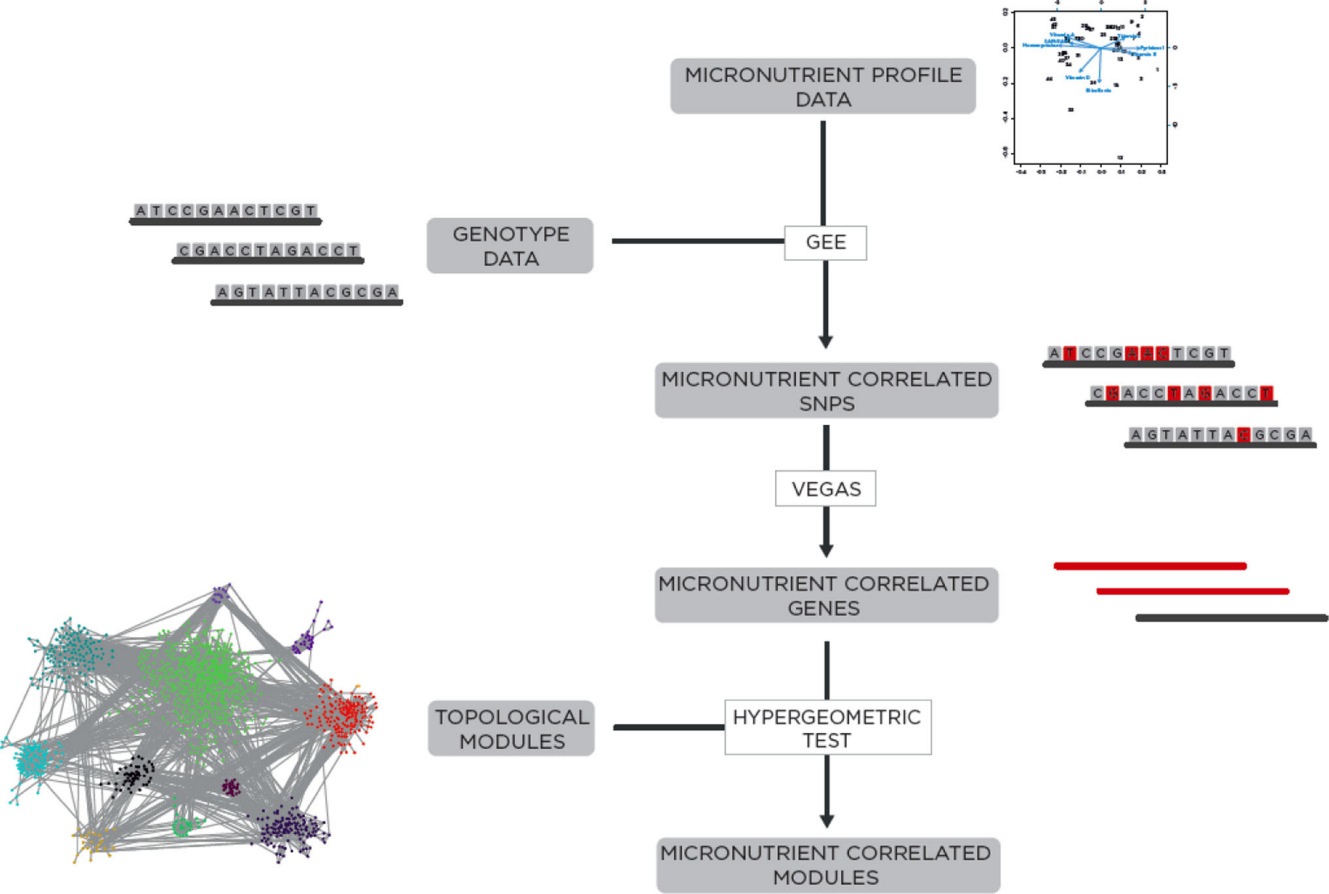
Methodological approach to the correlation of genotype and metabolite profile via network analysis. Metabolite data were first summarized by a single principal component and compared to each SNP (*gray boxes*) in the genetic dataset using GEE. Nominal *p* values from the GEE analysis (SNPs in *red boxes*) were used as input to the VEGAS algorithm to determine gene-level *p* values from SNP-level data (*red lines* indicate a gene with multiple SNPs, *black line* with single SNPs). Significant genes were then mapped to the global interaction network and considered as “hits” in hypergeometric tests of each topological module in the network

**Fig. 5:**
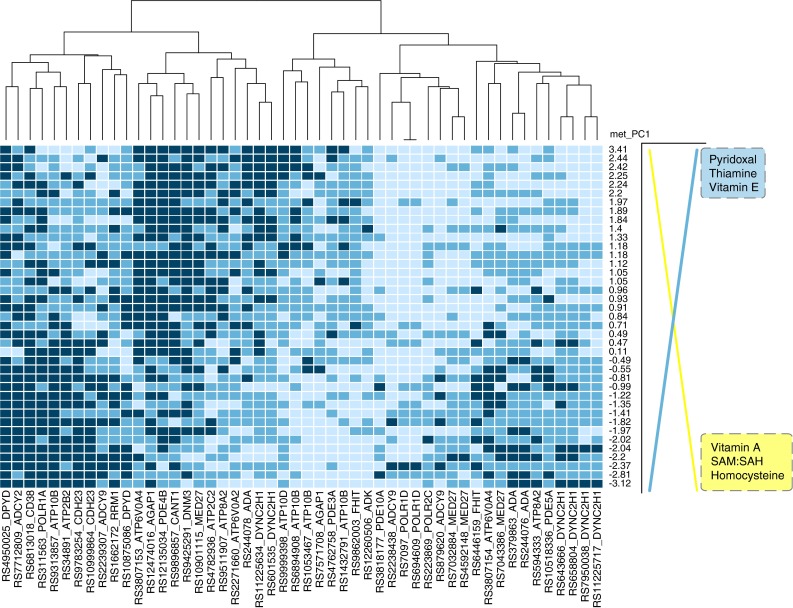
Genetic pattern for top 50 most significant SNPs (*y*-axis) in module 18 associated with Met_PC1 (*x*-axis)

The genes in the global interaction network collectively contained 125,959 SNPs represented on the 1 M Quad Array after preprocessing genotyping data. To associate the network to the experimental data, the met_PC1 was modeled as a function of genotype at each SNP locus using general estimating equations (GEE), controlling for age, sex, average Healthy Eating Index score, and sibling relationships among the participants (Fig. 4). A total of 3234 SNPs were significantly correlated with met_PC1 after correction for multiple testing (adjusted *p* < 0.05; Supplement 2). An example of the genotype results using the top 50 most significant SNPs is shown in Fig. 5, which shows differences in genotypes across met_PC1 values. The complex combination of all identified SNPs contributes to met_PC1 values, which are composed of 5 metabolites and SAM/SAH. Others have concluded that complex phenotypes result from the contribution of thousands of SNPs, each with low effect size (Stahl et al. 2012; Hemani et al. 2013).

Nominal *p* values were used as input for the VEGAS algorithm, which accounts for size, level of polymorphism, and linkage disequilibrium relationships within genes to determine genewise *p* values from SNP-level results (Liu et al. 2010). The result was 1,875 statistically significant genes associated with the met_PC1 variable, which were unevenly distributed among 46 of the 58 modules (Supplement 3).

Hypergeometric tests (see “Materials and methods” section) were used to test for significant overrepresentation of met_PC1 genes and met_PC1 proteins with *q* < 0.1 in each of the 58 modules. No modules were significantly enriched in met_PC1 proteins; however, four modules (Table 1) were found to be significantly enriched in genes related to the met_PC1 variable using a *q* value of <0.1 for the module. Module 45 had ten genes, of which four were significantly associated with met_PC1. These were the intracellular-localized proteins coproporphyrinogen oxidase (*CPOX*) and high-mobility group protein 1 (*HMG1*) and two plasma membrane-associated proteins sarcoglycan zeta (*SGCZ*) and sphingomyelin synthase 1 (*SGMS1*). Biological interpretation of this module is difficult as these 10 genes are significantly overrepresented in a set of functionally distinct pathways (porphyrin and chlorophyll metabolism, viral myocarditis, arrhythmogenic right ventricular cardiomyopathy, hypertrophic cardiomyopathy, dilated cardiomyopathy). Although the cardiac function pathways are potentially interesting, developing a strong hypothetical relationship between module function and nutritional health is difficult with such a small number of genes. Hence, the remaining discussion will focus on modules 52, 2, and 18. Each module was also tested for the enrichment of genes that interact with, metabolize, or are regulated by the metabolites measured in this study (Monteiro et al. 2014). These “micronutrient neighborhoods” (275 genes in total) were identified in the MetaCore database (version 6.10, build 31731) as all genes that directly interacted with metabolites measured in this study. The neighborhood may be considered a distinct level or subsystem between a pathway (e.g., the one-carbon pathway) and the modules within the global network. Understanding the entire system requires knowledge from the single reaction, pathway, neighborhood, and integrated network. Three modules of the 58 in the network were statistically enriched in these micronutrient genes (Table 2). Notably, module 18 was enriched in both the met_PC1 genes (Table 1), and the neighborhood genes (Table 2) linked to the metabolites measured in the study, thus indicating that it contains both statistical associations with micronutrient levels and also known functional associations with micronutrients.

**Table 1:** Network modules significantly enriched in met_PC1 genes

Module ID	Module size	Expected hits	Observed hits	*p* value	Adjusted *p* value
52	2,351	317.35	415	1.33 × 10^−10^	3.86 × 10^−9^
2	1,157	156.18	188	2.23 × 10^−3^	3.23 × 10^−2^
45	10	1.35	4	6.23 × 10^−3^	6.02 × 10^−2^
18	465	62.76	79	1.22 × 10^−2^	8.86 × 10^−2^

**Table 2:** Network modules significantly enriched in neighborhood micronutrient genes

Module ID	Module size	Expected hits	Observed hits	*p* value	Adjusted *p* value
47	580	9.47	62	8.23 × 10^−35^	2.39 × 10^−33^
18	465	7.6	42	9.07 × 10^−21^	1.32 × 10^−19^
11	11	0.18	10	1.74 × 10^−22^	1.68 × 10^−19^

#### Functional and genetic analyses of statistically significant genes and modules

In order to assess the biological processes that may be directly or indirectly implicated by genetic variation in our met_PC1 genes, the functional profile of each significant module was determined using data mining tools including the ClueGO plugin in Cytoscape (Bindea et al. 2009), the KEGG pathway database (http://www.genome.jp/kegg/pathway), ArrayTrack QTL (Xu et al. 2010) database, and literature mining. All pathways described in the ClueGO analysis results were significantly overrepresented in the given module (adjusted *p* value <0.05).

#### Module 18: Functional annotation

**Fig. 6:**
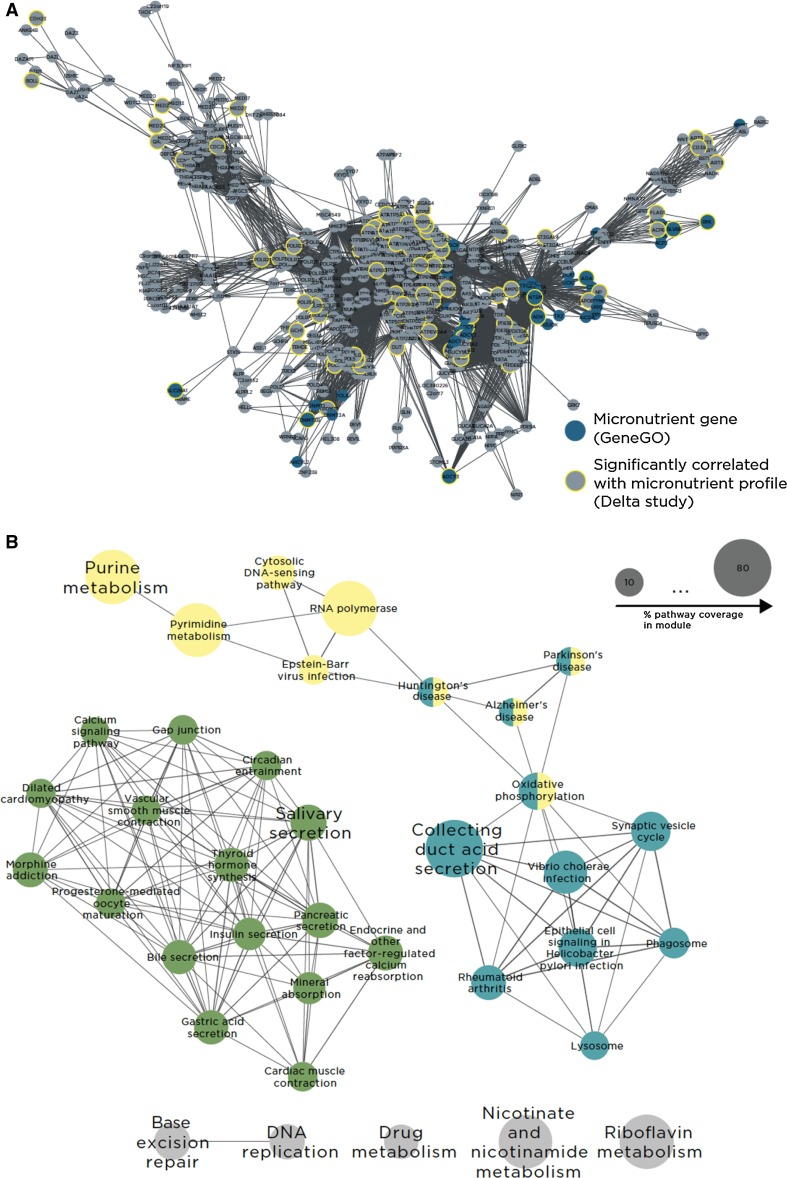
Detail image of module 18 (**a**), highlighting genes that are functionally linked to micronutrients (*blue nodes*) and genes that are statistically associated (via genotype) with micronutrient profile in the delta population (*yellow circles*). **b** Significantly overrepresented KEGG pathways in module 18, wherein each node represents a pathway, and the edges indicate the level of similarity between pathways based on shared gene content

Based on KEGG pathway annotations, the genes in Module 18 (Fig. 6) included about 80 % of the pyrimidine pathway genes and similar percentages for purine, nicotinamide, RNA polymerase, riboflavin, and collecting duct secretion processes (Supplement 3, Module column and Supplement 4). Parts of these pathways and processes may be found in other modules since the simulated annealing algorithm optimizes local interactions and does not consider the boundaries between pathways, as they exist in pathway databases.

**Fig. 7:**
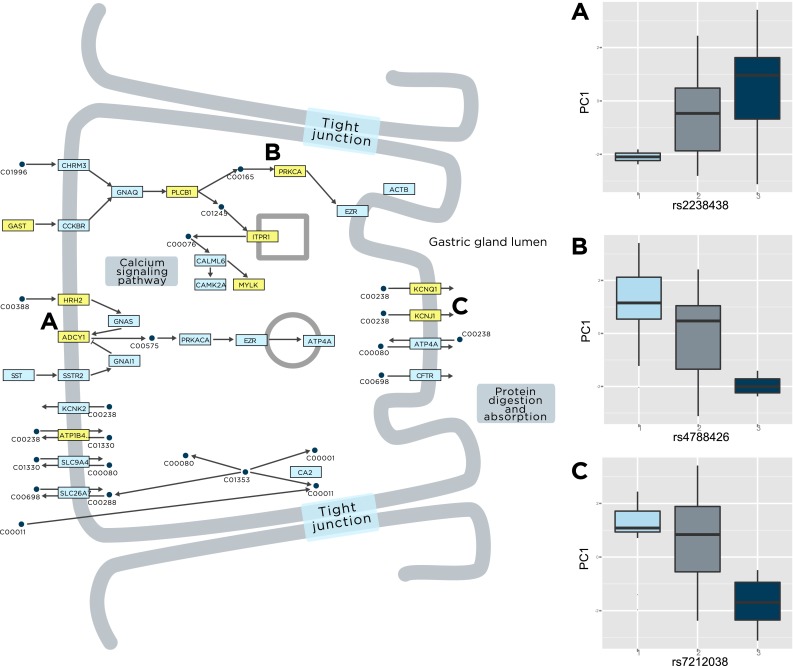
Genetic association of gastric acid secretion pathway genes with met_PC1. *Rectangles* represent pathway genes, and *circles* represent metabolites. Genes that were significantly correlated with met_PC1 are highlighted in *yellow*, and three representative example SNPs are shown in **a**–**c**

Graphical representation of the KEGG pathways in Module 18 provides a deeper view of the processes represented in this module (Fig. 6; Supplement 4, BarGraph18). Specifically, a cluster of Module 18 nodes includes genes that function in salivary, bile, pancreatic, and gastric acid secretion processes, such as *ADCY1*, *GAST,* and *KCNJ1*. Processes related to secretion functions, specifically gap junction structures, calcium and chemokine signaling pathways, and vascular smooth muscle contraction, are also strongly represented in this section of Module 18. Several pathways and networks involved in DNA repair processes are found in this module. The relationships of genes associated with met_PC1 with gastrointestinal function are illustrated the KEGG gastric acid secretion pathway shown in Fig. 7. Genes significantly correlated with met_PC1 are highlighted in yellow. Representative samples of SNP genotype differences in *ADCY1*, *PRKCA*, and *KCNJ1* are shown in the boxplots (Fig. 7a–c). These results suggest that genetic variation in these genes may cause dynamic variation in gut function, which may alter plasma micronutrient levels.

#### Module 2: Functional annotation

Module 2 is functionally enriched in immune function pathways and processes influenced by or involved in infectious diseases (Supplement 4, Barograph). Over 70 % of the genes involved in complement/coagulation pathways are found in this pathway. In addition, disease pathways affected by inflammation such as Alzheimer’s, type 1 diabetes, and rheumatoid arthritis are also represented in this module. Proteomic analysis of blood proteins demonstrated the association between a combination of metabolites including micronutrients (met_PC1) and inflammatory processes (Fig. 2).

#### Module 52: Functional annotation

Module 52 is the largest in the network with over 2300 genes of which 422 had SNPs statistically associated with met_PC1. Genes and pathways involved in immune functions are enriched in module 52 (Supplement 4, BarGraph52) with cytokine signaling and other immune pathways overlapping with Module 2. The secretory and absorption pathways in Module 18 also have components in Module 52. About 75 % of the phosphatidylinositol and inositol phosphate pathways involved in proliferation, survival, migration, and differentiation in different cell types including the development and regulation of B-lymphocyte and T-lymphocyte functions (So and Fruman 2012) are found in Module 52. Functional analysis also highlights the known links between diabetes and immune function, since type 1 and type 2 diabetes genes and pathways and ~50 % of the insulin signaling pathway occur in Module 52.

#### Quantitative trait loci mapping and cofactor analyses

**Fig. 8:**
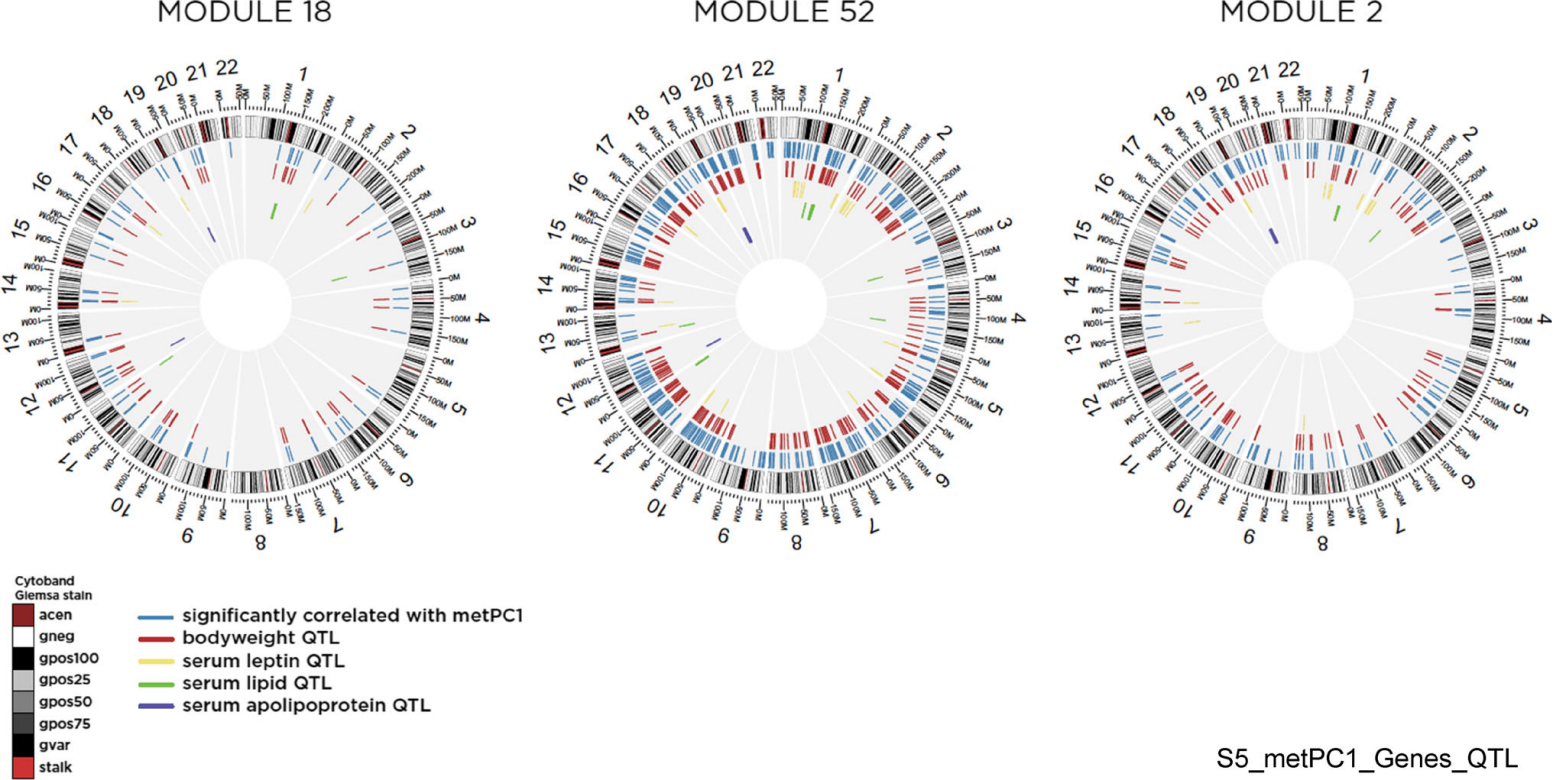
Circular genome images of modules 18, 52, and 2, each illustrating genes significantly correlated with met_PC1, and overlapping human QTLs associated with body weight, serum leptin, serum lipid, and serum apolipoprotein

The mapping to functional systems described above associates variation in met_PC1 with a wide range of biological processes consistent with the role of cofactors in enzymatic reactions, structural components of enzymes and proteins, and regulatory processes. Another approach to associate a gene with a phenotype is to determine the gene’s chromosomal location relative to genomic regions contributing to polygenic phenotypes identified by quantitative trait loci (QTL) and GWAS data (Kaput et al. 1994, 2004). In many cases, GWAS identify the most likely candidate genes within a region that might explain some aspect of the phenotype being studied. However, in the absence of direct genetic or biochemical experimental data, other genes in the QTL and GWAS regions may also contribute to the phenotype studied. Genes significantly correlated with met_PC1 were used as search terms in the ArrayTrack (Harris et al. 2009) QTL tool (Xu et al. 2010) to identify those that overlap with a 1M-bp region containing the QTL contributing to 36 phenotypes, selected based on physiological relevance to this study. A large number of met_PC1 genes overlapped with QTL for these 36 phenotypes; however, only QTLs for body weight, serum apolipoprotein E, serum leptin, and serum lipid levels were statistically enriched with these genes (*q* < 0.15). Met_PC1 genes mapping to these significant phenotypes are shown in Fig. 8 (listed in Supplement 5).

Lists of statistically significant genes alone, in modules, or mapped to QTLs identify potential candidate genes for a given phenotype. However, biological processes are necessarily controlled by gene–environment interactions. To associate the genes identified by data mining methods with nutrients, GeneCards and EBI’s cofactor database were searched for each of the genes mapping to QTLs for plasma levels of leptin, adiponectin, glucose, and for type 2 diabetes mellitus (T2DM) loci. Many of the statistically significant met_PC1 genes that mapped to these loci had a metal cofactor, and only a few required organic cofactors (not shown). For example, *CD320*, the transcobalamin receptor, mapped to the GLUCO3_H QTL (glucose level) on chromosome 19. *LRP2*, which is involved in vitamin uptake, mapped to a chromosomal region (GLUCO15_H on chromosome 2) associated with hyperglycemia. Several met_PC1 genes (*CHKA* which is involved in choline metabolism; *NOX4*, *TM7SF2*, *ALDH3B1*, *NDUFS8* are associated with NADPH) mapped to serum adiponectin level QTLs. Two genes (*SHMT1*, cofactors pyridoxal phosphate and folate; *ALDH3A2*–NADPH) mapped to serum leptin QTLs on chromosome 17. *DNMT3B* mapped to a serum cholesterol QTL and to a T2DM susceptibility locus on chromosome 20.

The limited ability to measure gene expression, protein levels, or enzyme activities in the appropriate tissues often prevents testing the contribution of a gene to a phenotype. Proteins in blood may be surrogates or involved in specific phenotypes. Eight of the 51 proteins correlated with met_PC1 (Fig. 2) mapped to 11 different QTLs (Table 3). While six of these proteins may be released from damaged cells for unknown reasons in the healthy state, two proteases normally found in the plasma were associated with body weight (KLK11, C1S), serum cholesterol (C1S), and APOE levels, blood pressure, and susceptibility to COPD (KLK11). Plausible biochemical explanations could be made for their participation in these phenotypes due to their enzymatic activities but further genetic and biochemical studies are necessary to test whether they are involved in these conditions.

**Table 3:** Met_PC1 correlated proteins mapping to quantitative trait loci (definitions)

Gene	Plasma cellular	BP	Body weight	Susceptibility					Serum levels			
				COPD	MI	MS	Prostate	Osteoarth	ApoE	Chol	Fibrin	Ghrelin
*C1S*	P		BW65							SCL46		
			BW81									
			BW82									
			BW192									
*CDC2*	C										FBRL4	
											FBRL5	
*CDH12*	P	BP47			MY4	MULTSCL29			SAPOA3			
					MYI12							
*CSNK2A1*	C		BW368				PRSTS150			SCL126		
							PRSTS436					
*KLK11*	P	BP69	BW89	COPD5					SAPOE1			
			BW139	COPD9								
*MAPK1*	C	BP71										
*PGAM1*	C		BW91							SCL34	FBRL4	
											FBRL5	
*PGD*	C	BP9		COPD17			PRSTS240	OSTEAR17				SGHRL1
		BP17										

Plasma and cellular refer to accepted location of protein

*BP* blood pressure, *COPD* chronic obstructive pulmonary disease, *MI* myocardial infarction, *MS* multiple sclerosis, *osteoarth* osteoarthritis, *chol* cholesterol, *fibrin* fibrinogen

## Discussion

Health and disease processes result from a complex interaction between multiple genes and environmental factors. The systems nutrition analyses reported here used data from dietary intakes, plasma and erythrocyte metabolite levels, plasma proteins, and genetic makeup in a cohort of children/teens aged 6–14. Discussed below are (1) the main biological results, (2) strategy and methodological considerations, and finally, (3) implications for health and disease research.

### Biological findings

#### Met_PC1 and SAM/SAH

Principal component analysis (PCA) identified a metabolite pattern, met_PC1, with positive and negative correlations between plasma micronutrients, plasma Hcy, and SAM/SAH ratio in erythrocytes. Plasma vitamin A and Hcy correlated positively with SAM/SAH, and vitamin E, thiamine, and pyridoxal correlated negatively in this population. While statistical associations do not prove causality, these correlations suggest that micronutrients and metabolites operate within a network that includes SAM/SAH metabolism. Altering the proportion of metabolites relative to each other may alter methylation potential and therefore epigenetic reactions. Others have shown that SAM/SAH correlated with differences in methylation at metastable epialleles based on season and food availability (Waterland et al. 2010). Changes in epigenetic programming at critical developmental windows such as in utero, early childhood, or during puberty have been associated with developmental plasticity, health, and susceptibility to chronic diseases in adults (Barker et al. 1993; Gluckman et al. 2009; Kussmann et al. 2010).

#### Met_PC1 and plasma proteins

Met_PC1 was also associated with levels of pro-inflammatory proteins. Individuals with high vitamin A, Hcy (but still below the clinical cutoff of 15 µmol/L), and SAM/SAH had lower levels of many of these inflammatory proteins. The correlation was modest for any single protein to met_PC1 value. However, certain proteins shared similar correlation coefficients and functional analysis based on gene ontologies, and some of these correlated proteins that participated in the same networks. Since met_PC1 is an empirically defined value specific to this study, the correlations among these plasma metabolites will necessarily require testing in other genetic makeups and environments.

#### Met_PC1 and global protein topological analysis

To discover whether the met_PC1 variable was associated with genetic variation in molecular interaction networks or subsystems, a metabolic/protein–protein interaction network was constructed based on two manually curated interaction databases (Ma et al. 2007; Yu et al. 2012). The network was partitioned into topological modules, each of which was assessed for significant enrichment with met_PC1-correlated genes using a hypergeometric test. Three modules were identified, 2 of which contained substantial numbers of immune and metabolic function genes and the third included genes in a range of secretory and gastrointestinal functions. Although the met_PC1 genes were not directly functionally annotated to every one of the identified processes/pathways in these modules, they may either be directly contained in these processes/pathways, or indirectly connected via a small number of degrees of separation. Variation in plasma micronutrient levels and Hcy, and erythrocyte SAM and SAH, and specifically the ratios of these metabolites relative to each other, was associated with genetic variations in immune and gastrointestinal functions. Chronic disturbance in gastrointestinal function (such as that seen in inflammatory bowel disease, Crohn’s disease, and environmental enteropathy) may directly contribute to micronutrient deficiencies due to altered nutrient absorption (Valentini et al. 2008). Although the cohort in the present study did not present with diagnosed intestinal disorders, it may be the case that a range of SNPs contributes to subclinical variation in gastrointestinal function, which then relates to variation in micronutrient levels. Additional focused work on enterocytes and intestinal immune cells would be required to clarify the potential functional consequences of the SNPs identified in this study.

Metabolite principal component 1 (Met_PC1) was derived from the relationship of vitamins A, E, thiamine, pyridoxal, and the metabolites Hcy, SAM/SAH ratio. Correlations of met_PC1 to immunity were not unexpected since a rich literature exists for individual micronutrients and various aspects of immune function and regulation (Bhaskaram 2002; Maggini et al. 2007; Baeke et al. 2010; Ströhle et al. 2011; Ooi et al. 2012). The genetic analysis nevertheless revealed new insights into the many genes and their functions that may be associated with different plasma levels of metabolites and therefore with diet.

### Strategy and methodological considerations

The experimental design included several uncommon approaches for human nutritional studies:

1. The use of CBPR with biomedical and network biology analyses. Community-based research engages participants in the research study and provides opportunities for health- and nutrition-related exchanges between community members and researchers. Community-based research is done in “real” time with lifestyle and other environmental conditions that are not under the control of the researcher. These factors likely introduce noise into the study and analyses, but the measured biological “signals” include the contribution from those unmeasured influences. Our goal was to measure as many physiological and environmental variables as possible to associate signals with phenotype. In addition, community-based results from such studies are likely to be translated more rapidly to individuals and populations (McCabe-Sellers et al. 2008).
2. Data from this study were previously analyzed at the group level (such as between SAM/SAH groups) and at the population level (Monteiro et al. 2014). We have also extensively analyzed dietary intake patterns, metabolomics, proteomic, and genomic data for individual participants in this study. For example, dietary intake variables were compared to metabolite patterns in each participant to determine whether common patterns could be identified at the individual level, and DNA ancestry was analyzed for each individual for the possibility of using genetic admixture mapping methods (Cheng et al. 2010) (data not shown). Methods which identify groups of individuals with related metabolic features but still allow for n-of-1 analysis may extend the recent personal omics analysis for molecular and medical phenotypes (Chen et al. 2012). Reporting data and results from studies with more than one individual, however, may require development of novel publication strategies.
3. Levels of metabolites in each participant were analyzed and shown in one figure as opposed to reporting results of the average metabolite level in separate graphs. Although such methods are common in transcriptomic and metabolomics literature, we identified patterns of metabolite levels that revealed unanticipated nutrient–nutrient statistical interactions. Standard PCA converted the graphic representation of metabolite levels to a statistic specific called metabolite principal component 1 (met_PC1). Met_PC1 represented 6 strongly and one weakly associated (vitamin D) measured metabolites and their interactions. Although the value of this statistic is specific for the study reported here, similar methods may allow for more comprehensive analyses of interacting metabolites.
4. Analyzing genetic differences based on met_PC1 in a metabolic/PPI network partitioned into topological modules allowed for the identification of physiological functions (immune, metabolic, and secretory) associated with gene–metabolite relationships identified in our statistical analysis. Rather than seeking a small number of SNPs with large effect on our phenotype, our network-based approach inherently highlights multivariate groups of functionally related SNPs/genes that are statistically associated with a phenotype. Defined interventions can be developed from our results and, equally importantly, tested by measuring parameters of immune and GI function that were identified in this study.

Although the limiting aspect of the study reported here is the small sample size (45 genetically unique individuals with 61 sets of metabolite, protein, and diet variables), the combination of these methodological approaches may provide new strategies for genomic and nutrigenomic studies. Expanded sample size will be particularly important for genetic analysis in order to avoid exceedingly small sample sizes for minor alleles, as we observed at times in our genetic analysis.

### Implications for reproducibility

The results described in this manuscript and recent publications on intra-individual variability in physiological status found in environments that have large changes in nutrient availability (Dominguez-Salas et al. 2013) demonstrate the difficulty in replicating biomedical research, particularly for genetic and gene–environment interaction associations. Hierarchical clustering of proteins (Fig. 2) and SNPs (Fig. 5) correlated with met_PC1 helps visualize the proteomic and genotypic differences as combinations of SNPs or proteins rather than as single markers (even though these were derived from univariate analysis and corrected for multiple comparisons). No single protein or SNP is always correlated with met_PC1. This is what we would expect to observe, in part because of gene–gene and gene–environment interactions, epigenetic regulation, and other interactions. This perspective better fits the biological reality of multiple genes and their products contributing to a complex phenotype (in this case met_PC1). Our working hypothesis is that testing these associations in other populations and also in others experimental designs would be needed to identify common patterns of variants within these genotype data sets that might explain the percentage of genetic contribution to a given phenotype. Subsets of genes will contribute or not contribute to a complex phenotype (e.g., obesity and diabetes) based on interactions with diet or other environmental factors. In addition, the adaptations to diverse environments over human evolution may have selected different collections of genes for similar environments in different environments. The most notable, and still controversial example, is the different functional adaptations to Tibetan and Andean high altitudes (Beall 2007). Nevertheless, we predict that gene–environment interactions producing the same phenotype will have overlapping genes (much like Venn diagrams). Some pathways and therefore genes will be shared, and others may contribute less significantly in different genetic subpopulations. Discovering these similarities and differences may lead to an understanding of targeting diet and lifestyles to optimize health.

## Electronic supplementary material

Below is the link to the electronic supplementary material.

Proteins significantly correlated with met_PC1 (corrected p value < 0.1). (**Figure 2**) (XLS 85 kb)

Significant SNPs associated with Met_PC1. (XLSX 390 kb)

Genewise analysis of SNPs associated with met_PC1 (**Figure 6**) (XLSX 425 kb)

Cytoscape functional analysis of modules 2, 18, and 52 (PPTX 546 kb)

Met_PC1 genes mapping to quantitative trait loci (**Figure 8** ) (XLSX 1090 kb)
