# Supplementary material for: Genetic associations with micronutrient levels identified in immune and gastrointestinal networks
Source: Genes Nutr. 2014 May 31;9(4):408. doi: 10.1007/s12263-014-0408-4 (PMC4169061; doi:10.1007/s12263-014-0408-4)
Supplement: Supplementary file 4 — Cytoscape functional analysis of modules 2, 18, and 52 (PPTX 546 kb) [file 12263_2014_408_MOESM4_ESM.pptx]

## Slide 1
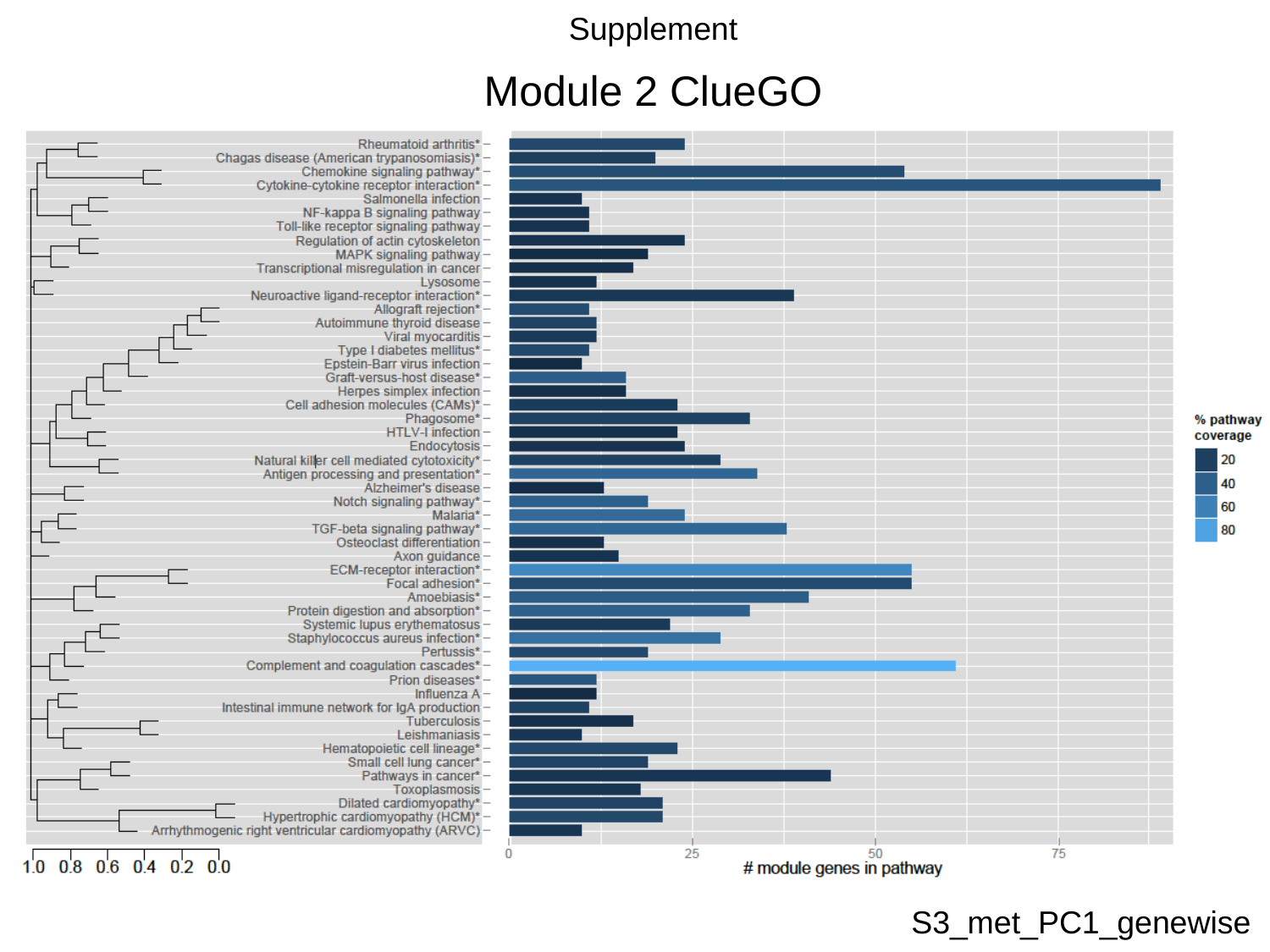

Supplement
Module 2 ClueGO
S3_met_PC1_genewise

## Slide 2
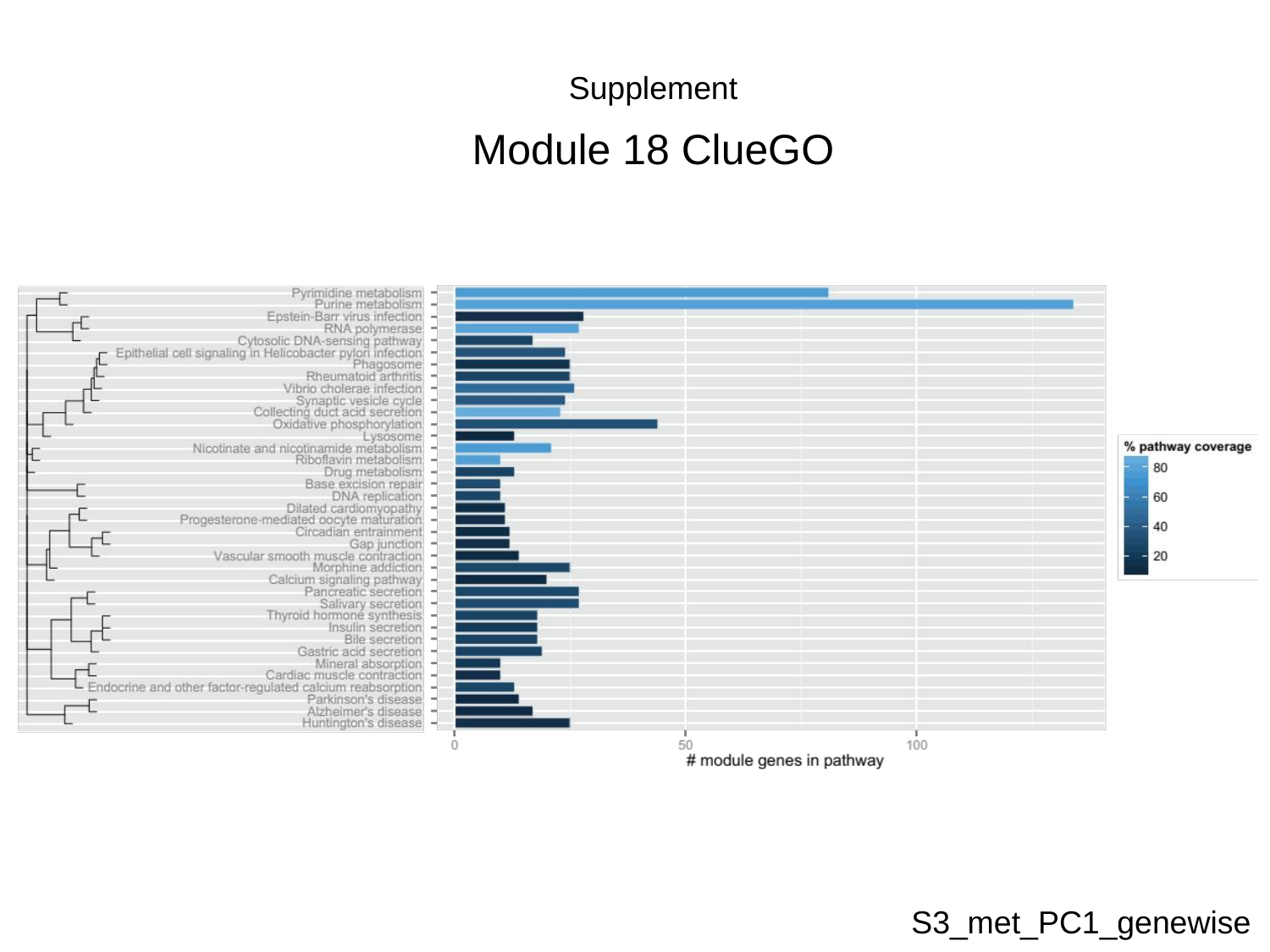

Supplement
Module 18 ClueGO
S3_met_PC1_genewise

## Slide 3
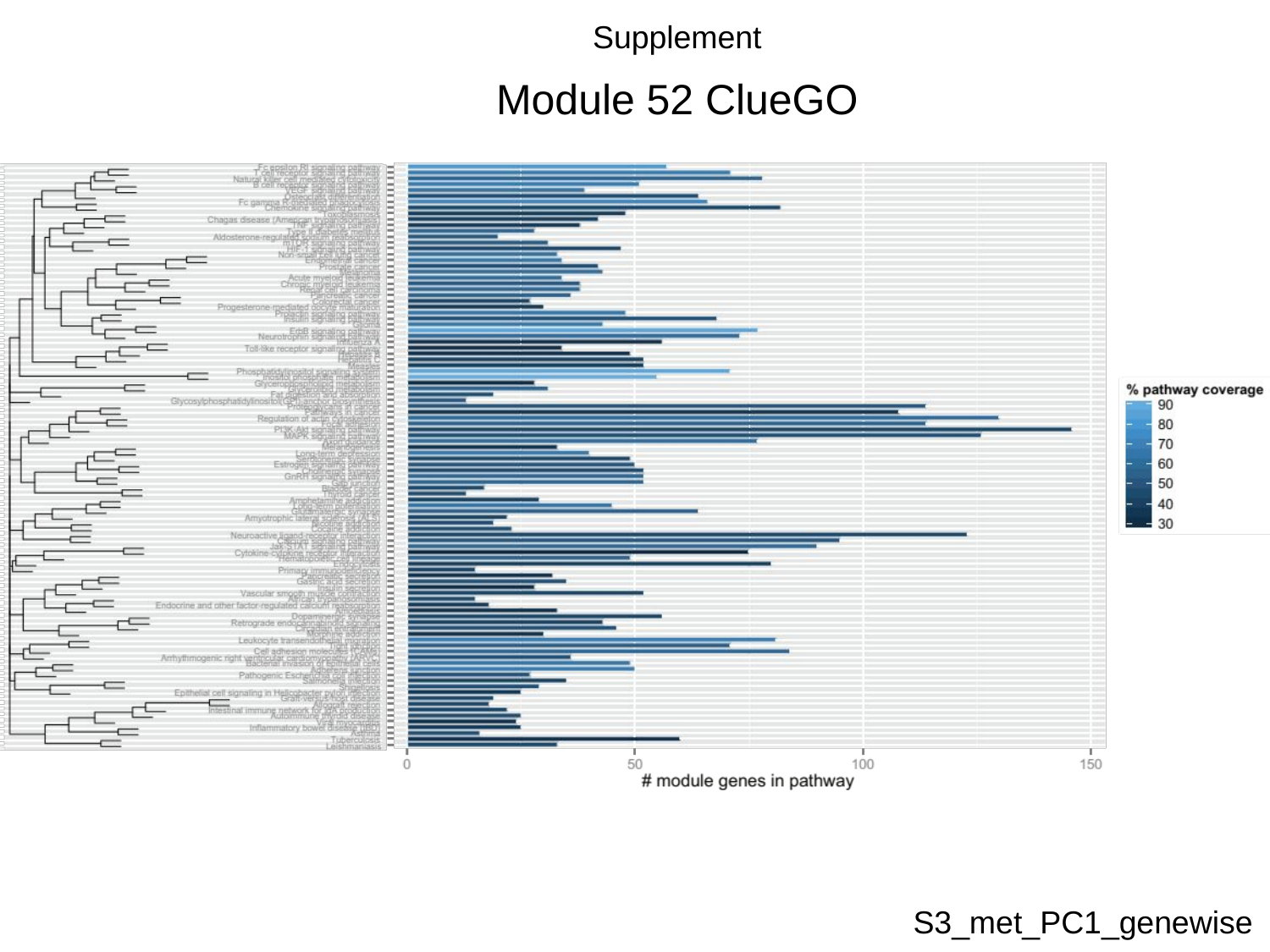

Supplement
Module 52 ClueGO
S3_met_PC1_genewise
